# The Unified Efficiency Evaluation of China’s Industrial Waste Gas Considering Pollution Prevention and End-Of-Pipe Treatment

**DOI:** 10.3390/ijerph17165724

**Published:** 2020-08-07

**Authors:** Yanhong Tang, Yingwen Chen, Rui Yang, Xin Miao

**Affiliations:** 1 School of Public Administration and Law, Northeast Agricultural University, Harbin 150030, China; tangyanhong@neau.edu.cn; 2 School of Management, Harbin Institute of Technology, Harbin 150001, China; 18b910068@stu.hit.edu.cn (R.Y.); miaoxin@hit.edu.cn (X.M.)

**Keywords:** network data envelopment analysis, pollution prevention, end-of-pipe treatment, industrial waste gas, grey relation analysis

## Abstract

With the deepening of industrialization and urbanization in China, air pollution has become the most serious environmental issue due to huge energy consumption, which threatens the health of residents and the sustainable development of the country. Increasing attention has been paid to the efficiency evaluation of industrial system due to its fast development and severe air pollution emissions, but the efficiency evaluation on China’s industrial waste gas still has scope for improvement. This paper proposes a global non-radial Network Data Envelopment Analysis (NDEA) model from the perspective of pollution prevention (PP) and end-of-pipe treatment (ET), to explore the potential reduction of generation and emission of air pollutants in China’s industrial system. Given the differences of different air pollution treatment capacities, the ET stage is further subdivided into three parallel sub-stages, corresponding to SO_2_, NO_X_, and soot and dust (SD), respectively. Then, grey relation analysis (GRA) is adopted to figure out the key factor affecting the unified efficiency. The main findings are summarized as follows: firstly, the unified efficiency of China’s industrial waste gas underperformed nationwide, and most provinces had the potential to reduce the generation and emission of industrial waste gas. Secondly, the PP efficiency outperformed the ET efficiency in many provinces and the efficiency gap between two stages increasingly narrowed except in 2014. Thirdly, the unified efficiency in the eastern area performed well, while the area disparities increased significantly after 2012. Fourthly, significant differences were found in three ET efficiencies and the ET efficiency of NO_X_ was higher than that of SO_2_ and SD in the sample period. Finally, the results of GRA indicated that different air pollutants had distinct influence on the improvement of the unified efficiency in three areas. To promote the unified efficiency of industrial waste gas, some pertinent policy suggestions are put forward from the perspectives of sub-stages, air pollutants and areas.

## 1. Introduction

**Figure 1:**
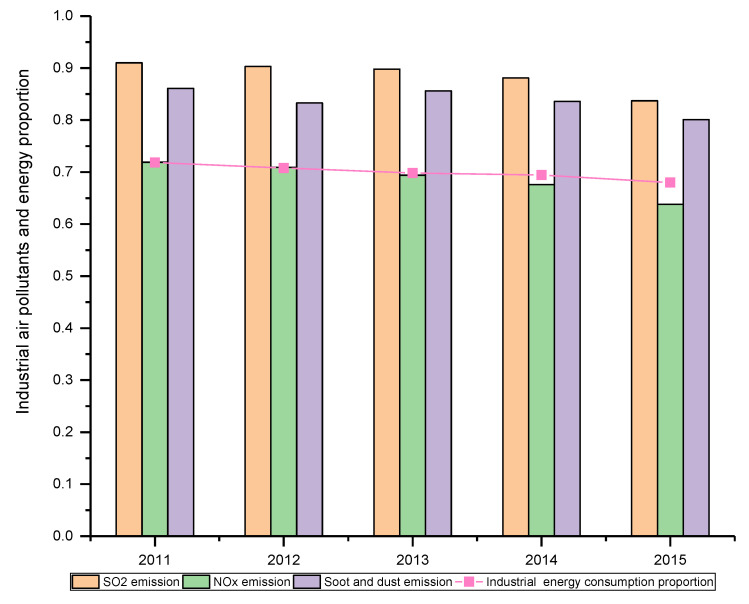
Proportion of industrial waste gas.

In the past decades, China’s economy has achieved remarkable achievements. Its gross domestic product (GDP) exceeds 90 trillion RMB in 2018 [1], which is 244.8 times compared with that of 1978. The industrial sector plays an important role in China’s economic development [2] but at the cost of heavily energy consumption and pollutant emissions. Although China’s industrial structure optimization and energy structure adjustment have achieved some accomplishments in recent years, which can be verified from the declining industrial energy consumption proportion as shown in Figure 1, the energy consumption of the industrial sector still reached 2944.88 million tons of standard coal in 2018 [1], accounting for 63.5% of the total energy consumption. Increasingly serious air pollution caused by the great energy consumption has made the life expectancy decrease by 5.5 years [3]. China has been considered as one of the countries with the most heavy air pollution in the world [4], as its environmental performance index ranked 120 out of 180 countries, of which air quality index ranked 177 [5]. Figure 1 further shows the emission proportion of three industrial air pollutants (SO_2_, NO_X_, and soot and dust (SD)) in China during the period of “12th Five-Year Plan” (2011–2015) [6]. It can be found that the industrial sector contributes most of the pollutant emissions, especially for SO_2_ and SD, which are main causes of the acid rain and human respiratory diseases. Currently, achieving the reduction of generation and emission of industrial waste gas has become more and more urgent so as to improve air quality and promote sustainable development in China.

Air pollutants such as SO_2_ and SD, which can cause serious cardiopulmonary diseases, not only threaten people’s health [7], but also pose a great threat to China’s sustainable development, for example, dust and fume has a great negative impact on the healthy development of agriculture, industry and transportation industry [8]. Among the aggravating environmental pollution, air pollution treatment has become China’s top environmental issue [9]. Some scholars have evaluated the China’s air quality, including [3,4,10,11], however, these studies can only obtain an overall efficiency value, ignoring the differences in the generation and treatment capacity of different air pollutants among provinces which cannot truly reflect the industrial pollution situation. Taking 2015 as an example, the generation of three kinds of air pollutants (SO_2_, NO_X_, and SD) in Beijing and Xinjiang were 64,153 tons, 52,233 tons, and 1,461,648 tons, 1,893,615 tons, 670,150 tons, and 21,986,103 tons respectively, while the corresponding investments in pollution treatment were 157.79 million RMB, 1418.66 million RMB, and 212.41 million RMB, 1141.80 million RMB, 668.47 million RMB, and 1039.94 million RMB [12]. On the one hand, great differences can be found in the generation and treatment of different air pollutants for the same province. On the other hand, for different provinces, due to the diverse industrial structure and economic development level, there are also significant differences in the generation and treatment for the same pollutant. In this case, ignoring the obvious differences of different air pollutants may causes difficulties to the precise prevention and control of air pollution.

In addition, to reduce the damage of air pollution to social development and people’s health, the Chinese government has taken a series of measures which can be roughly divided into two categories. One is the end-of-pipe treatment (ET) method, such as various air pollution treatment technologies including desulfurization, denitrification and dedust. That is, after the generation of pollutants, they are treated by corresponding technologies to meet the national or industrial emission standards. The other is front-end prevention method and the energy efficiency improvement and energy structure adjustment (such as clean energy development) [13,14]. Although many scholars have pointed out that China’s energy efficiency has large room for improvement, how to achieve this improvement is always controversial. Some scholars believed that it is insufficient to make the production technology effective only by changing the input in the short term [15,16]. In contrast, energy structure adjustment seems to be a more sustainable way.

In fact, during the “11th Five-Year Plan” (2006–2010), the Chinese government has worked on adjusting the energy structure and reducing fossil fuel consumption [17]. Great achievements have been made since “12th Five-Year Plan” (2011–2015). Specifically, the consumption of coal and crude oil decreased gradually, from 77.8% and 8.5% in 2011 to 69.3% and 7.2% in 2018 [1]. In the same period, the consumption proportion of natural gas, primary electricity and other energy increased significantly, from 4.1% and 9.6% to 5.5% and 18.0%, respectively. Moreover, the Chinese government proposed the strategic goal of dual control of total energy consumption and energy consumption intensity during the “13th Five-Year Plan” (2016–2020). The reduction of fossil energy consumption is bound to bring about sharp reduction of air pollutants [18]. The way of reducing pollutants by reducing the energy can be regarded as the pollution prevention (PP) method. It is evident that both pollution prevention and end-of-pipe treatment are common in the current China’s industrial production, but there is no comprehensive research that considers them from the efficiency aspect.

Efficiency evaluation is an important way for an organization to better understand the past accomplishments and make suitable plans for the future [19]. As a non-parametric method, data envelopment analysis (DEA) has been widely applied to efficiency assessment in the fields of insurance industry [20], bank [21], transportation industry [22], high-tech industry [23] and so on due to its advantages. For example, DEA does not need to consider explicit relationships between inputs and outputs [24], and it can handle multiple inputs and outputs at the same time. Air quality evaluation based on DEA has also attracted much attention of scholars, such as [3,4,10,11]. As mentioned, however, both the significant differences of different air pollutants and the different pollution treatment methods (PP and ET) of industrial pollutants in China have often been ignored. To close the gap, this paper proposes a network DEA model from the perspective of PP and ET. On this basis, the end-of-pipe treatment is further divided into three parallel sub-stages, corresponding to SO_2_, NO_X_ and SD, respectively, to evaluate the corresponding efficiency of different air pollutants. Then, grey relation analysis (GRA) is used to figure out the key factor affecting the unified efficiency so that decision makers can maximize the effect of limited resources. Compared with previous studies, the contributions of this paper are mainly reflected in two aspects: one is to comprehensively consider the front-end prevention and terminal treatment of air pollutants, which is more in line with the actual situation and helpful to accurately assess the current air quality of China’s industrial sector; The second is to consider the differences in the generation and treatment capacity of air pollutants in different provinces, which is conducive to analyze the end-of-pipe treatment of China’s industrial sector in detail.

The remainder of this paper is organized as follows: Section 2 reviews the associated literature of China’s industrial sector and presents the research gaps. Section 3 proposes the global non-radial network DEA model from the perspective of pollution prevention and end-of-pipe treatment and introduces the procedure of GRA. Section 4 presents the empirical analysis and Section 5 gives the conclusions, policy implications, and possible research directions in future.

## 2. Literature Review

Stochastic frontier analysis (SFA) and DEA are two main frontier analysis approaches, which have become increasingly popular to measure efficiency of different fields. The former is a parametric method, calculating efficiency through a prior functional form [25], which can account for the influence of random factors on output [26]. However, incorrect functional form may lead to inaccurate results. Moreover, this method is not suitable for the situation with multiple outputs [27]. By comparison, DEA is a non-parametric method without setting the production function form subjectively. And the method can deal with multiple outputs, which makes it widely used for different efficiency evaluations as mentioned above.

The energy efficiency, environmental efficiency, and eco-efficiency of industrial sector are always research hot points in past decades. Shi et al. [18] used DEA to examine the energy efficiency of industrial sectors in 28 provinces of China from 2000 to 2006 and found that the energy efficiency of the eastern area was significantly higher than that of the central and western areas. Taking industrial wastewater, waste gas, and solid waste as pollutants, Zhou et al. [28] developed a new slack-based measure (SBM) model considering energy and pollutants weight preference. They investigated the environmental efficiency of seven industrial sectors in China and claimed that the environmental efficiency was quite different for different industrial sectors. Meng et al. [29] proposed a non-radial DEA model to evaluate the environmental performance of industrial sectors of 30 provinces from 1998 to 2009 and proved that the environmental efficiency increased by 58% in the study period. Wang et al. [30] analyzed the eco-efficiency of 22 industrial sectors with similar pollutants through the hybrid super-efficiency SBM model, and further explored the ineffectiveness of each input-output index. Focusing on the heterogeneity of indicators, Wu et al. [31] assessed the energy and environmental efficiency of 38 industrial sectors in China by taking volatile hydroxy-benzene, cyanide, chemical oxygen demand, petroleum, and ammonia nitrogen as pollutants. The results indicated that the efficiency of each sector was low and great differences were found among sectors. Regarding COD, SO_2_, soot, dust, and solid waste as pollutants, Zhang et al. [32] examined the technical efficiency, environmental efficiency, and eco-efficiency of industrial sectors in 30 provinces, and found that for most provinces, the technical efficiency was higher than the environmental efficiency.

Previous studies regard the industrial production process as a black box, neglecting the internal structure, which cannot find out the internal reasons that lead to the inefficiency of the system [33]. With the proposal of network DEA [34], it becomes more popular in the efficiency evaluation of industrial sector. Dividing the industrial system into two sub-stages including production and treatment, Bian et al. [35] constructed a network SBM model to analyze the China’s industrial system, and verified that the efficiency of production sub-stage is much higher than that of treatment sub-stage. Wu et al. [36] calculated the total factor energy efficiency of China’s industrial system with the similar two stage division, and the result showed that the energy efficiency increased in the study period, and the efficiency in the production sub-stage was higher than that of treatment sub-stage. Based on the cooperative and non-cooperative strategy, Wu et al. [37] investigated China’s industrial production and pollution treatment efficiency in 2010. Taking the integrated utilization of industrial solid waste as the feedback index of wo sub-stages, Ding et al. [38] computed the industrial circular economic efficiency of 41 cities in the Yangtze River Delta, and they suggested that the average treatment efficiency was less than half of the production efficiency.

These network DEA models can find out the internal causes of system inefficiency from the perspective of sub-stages, which is conducive to find the weak links of the industrial system. However, due to the differences in pollution treatment capacity of different provinces, it is impossible to further figure out the specific causes of sub-stage inefficiency. For example, in the above two-stage industrial analysis, most of the studies believe that the treatment efficiency has a large improvement room [35,38], but it is uncertain which kind of pollutant (waste water, waste gas or solid waste) has insufficient treatment capacity, resulting policy implications may be biased. To address this issue, some scholars began to focus on specific pollutants under the network structure. Zhao et al. [39] divided the industrial water system into water resource utilization sub-stage and water pollution treatment sub-stage and evaluated the efficiency of 30 provinces in China from 2001 to 2014. Ding et al. [40] divided the industrial system into industrial production and wastewater treatment and examined the water-energy relationship of industrial sectors in China from 2011 to 2015. Considering only part of the industrial solid waste is treated in current period, the remainder is stored for later treatment. Tang et al. [41] used the dynamic network SBM model to evaluate the generation and treatment efficiency of industrial solid waste of 30 Chinese provinces during 2011–2015. Taking industrial waste gas as the research object, Li et al. [13] adopted the dynamic network SBM model to evaluate the production and waste gas treatment efficiency of China’s industrial sector in 2013–2016. Moreover, on the basis of dividing the industrial system into production and treatment, Shao et al. [2] subdivided the treatment sub-stage into waste water treatment and waste gas treatment, and investigated the eco-efficiency of 36 industrial sectors in China from 2007 to 2015.

Despite the large number of DEA papers on the China’s industrial system, both of them ignored the potential relationship between energy consumption and pollutants. As inevitable by-products in the industrial production, scholars have put forward different methods to deal with these pollutants, such as taking the pollutant as input [42], making linear transformation [43], and weak disposability [44]. Among them, weak disposability has been widely applied in industrial system research, such as [2,29,35,36,38]. There are two explanations for weak disposability. One is that it is feasible to reduce the desirable output with the same proportion as the undesirable output. For example, in thermal power plants, a 10% reduction in sulfur dioxide emission is possible if accompanied by a 10% reduction in electricity generation [45]. Another explanation is that when pollution reduction is the primary task, some neutral inputs (such as labor and capital) can be converted to deal with pollutants, so that the desirable output and the undesirable output decline at the same time [46,47]. It can be found that no matter which explanation ignores the potential relationship between energy input and pollutants. That is, reducing energy consumption, the corresponding pollutants should also be reduced [18]. This potential relationship reflects an idea of pollution prevention, which means reducing the generation of pollutants from the source. Although China has committed to cutting down the use of fossil energy in the “11th Five-Year Plan”, there is no comprehensive research on pollution prevention and end-of-pipe treatment from the aspect of efficiency. In addition, due to the different industrial structure and economic development level, the generation and treatment capacity of different air pollutants are also different in each province. Therefore, it is necessary to analyze the treatment efficiency of different pollutants separately.

In reality, decision makers not only care about the efficiency evaluation, but also pay close attention to the improvement of the inefficiency. Therefore, it is necessary to figure out the key factor affecting the efficiency, which is conductive to determine the improvement direction of DMUs (Decision making units, indicating industrial system in this paper). As an important part of grey theory, GRA is suitable for analyzing complicated interrelationships between multiple factors [48,49]. Specifically, GRA judges the degree of connection between different sequences according to the geometric correspondence between factors, which has been widely applied into the field of the green remanufacturing [50], the supplier selection [51], and so on. Some innovative research combining DEA with GRA has also been carried out. For example, Li et al. [52] constructed a generalized three stage DEA model to measure the innovation efficiency of semiconductor industry in China and used GRA to find the influencing factor of innovation efficiency. Yu et al. [53] adopted zero-sum-gains DEA model and GRA to explore the driving factors of carbon emission. Referring to these studies, GRA is also utilized in this paper to explore the key factors affecting the unified efficiency.

In short, the general framework of this paper is as follows. A global non radial network DEA model was first constructed from the perspective of pollution prevention and end-of-pipe treatment, focusing on exploring whether there is room for improvement in the generation and emission of industrial waste gas in China. On this basis, the end-of-pipe treatment stage was further subdivided into three parallel sub-stages corresponding to SO_2_, NO_X_, and SD, respectively. Then, the GRA was used to figure out the key factor influencing the unified efficiency so that decision maker could maximize the effect of limited resources to improve the unified efficiency.

## 3. Model Construction and Solution

**Figure 2:**
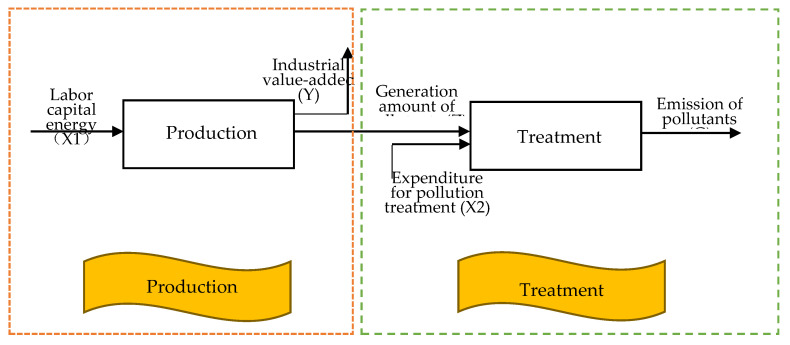
Two-stage structure of industrial system.

**Figure 3:**
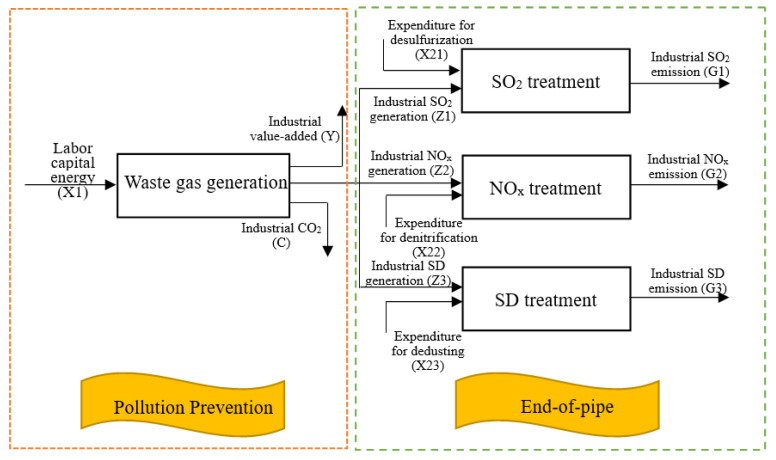
Pollution prevention and end-of-pipe treatment of industrial system. SD indicates Soot and Dust.

Figure 2 shows the general two-stage structure of industrial system, which has been widely used in efficiency research of industrial sector, such as [36,39,40]. However, the potential relationship between energy consumption and pollutants has been ignored in the previous studies. Considering the differences of air pollutant treatment capacities in different provinces, Figure 3 gives our new network structure model. The obvious difference between Figure 2 and Figure 3 is that the latter subdivides the end-of-pipe treatment into three parallel sub-stages. In addition, the essential distinction is that we take into account the potential relationship between energy consumption and pollutants in the production stage, which will be embodied in the following model. As this paper focuses on whether there exists reduction potential of the generation and emission of air pollutants in China’s industrial sector, we name the two sub-stages PP and ET, respectively. Next, the production technologies of different sub-stages are constructed in turn.

### 3.1. Pollution Prevention Technology

Assuming there are *n* DMUs, denoted as ${\mathrm{DMU}}_{j}(j=1,\ldots ,n)$. Each DMU has the network structure shown in Figure 2. In pollution prevention sub-stage, the input is divided into two categories including neutral input and non-neutral input. The former is denoted as $X_{i1j}(i1=1,\ldots ,m)$, mainly consisting of labor and capital, while the later indicates energy, denoted as $e_{j}$. Industrial value-added is regarded as the only desirable output, denoted as $Y_{j}$. The generations of SO_2_, NO_X_, and SD are the corresponding undesirable outputs, denoted as $Z_{pj}(p=1,2,3)$. CO_2_ is another inevitable pollutant since it contributes the most to global warming, denoted as $C_{j}$. There is no specific calculation method of the generation of CO_2_, referring to Shao et al. [2], the CO_2_ emission is included in this stage.

The traditional production technology (T) is shown as Equation (1):

(1) $$\begin{array}{l} T=\{(X1,e,Y,Z,C)|\\ \sum_{j=1}^{n}\lambda_{j}^{1}X_{i1j}\leq X_{i1},\;i1=1,\ldots ,m\\ \sum_{j=1}^{n}\lambda_{j}^{1}e_{j}\leq e\\ \sum_{j=1}^{n}\lambda_{j}^{1}Y_{j}\geq Y\\ \sum_{j=1}^{n}\lambda_{j}^{1}Z_{pj}\leq Z_{p},\;p=1,2,3\\ \sum_{j=1}^{n}\lambda_{j}^{1}C_{j}\leq C\\ \lambda_{j}^{1}\geq 0\} \end{array}$$

Here $\lambda_{j}^{1}$ is the intensity vector used to connect different input-output indexes. The traditional production technology conforms to some properties, such as the standard convexity and free disposability. Production technology (1) can meet different research needs by setting different objective function forms. However, the traditional production technology ignores the potential relationship between energy consumption and pollutants. Since the pollutants mainly come from the huge consumption of fossil energy, reducing energy consumption, the corresponding pollutants should also be reduced [18]. For this reason, Ray et al. [47] give the specific formula of cost disposability in the black box framework for the first time. The production technology with cost disposability ($T^{C}$) can be shown in Equation (2):

(2) $$\begin{array}{l} T^{C}=\{(X1,e,Y,Z,C)|\\ \sum_{j=1}^{n}\lambda_{j}^{1}X_{i1j}\leq X_{i1},\;i1=1,\ldots ,m\\ a\sum_{j=1}^{n}\lambda_{j}^{1}e_{j}=e\\ \sum_{j=1}^{n}\lambda_{j}^{1}Y_{j}\geq Y\\ a\sum_{j=1}^{n}\lambda_{j}^{1}Z_{pj}=Z_{p},\;p=1,2,3\\ a\sum_{j=1}^{n}\lambda_{j}^{1}C_{j}=C\\ \lambda_{j}^{1}\geq 0\} \end{array}$$

Cost disposability means that pollutants will reduce with the decrease of energy consumption, which is embodied by the reduction ratio a (0≤a≤1). However, the cost disposability requires all DMUs to be reduced at the same ratio, which is unreasonable since different DMUs have different production technology and external environment. Our paper presents a new form of cost disposability, corresponding production technology is denoted as $T^{NC}$, which allows different DMUs to reduce different reduction ratio, as shown in Equation (3):

(3) $$\begin{array}{l} T^{NC}=\{(X1,e,Y,Z,C)|\\ \sum_{j=1}^{n}\lambda_{j}^{1}X_{i1j}\leq X_{i1},\;i1=1,\ldots ,m\\ a_{j}\sum_{j=1}^{n}\lambda_{j}^{1}e_{j}=e\\ \sum_{j=1}^{n}\lambda_{j}^{1}Y_{j}\geq Y\\ a_{j}\sum_{j=1}^{n}\lambda_{j}^{1}Z_{pj}=Z_{p},\;p=1,2,3\\ a_{j}\sum_{j=1}^{n}\lambda_{j}^{1}C_{j}=C\\ \lambda_{j}^{1}\geq 0\} \end{array}$$

The new production technology not only considers the potential relationship between energy consumption and pollutants, but also allows different DMUs to reduce different ratio, which is more in line with the actual production process. It should be noted that no scholar has ever linked the cost disposability with the idea of pollution prevention, which can be regarded as one of the innovations of this paper.

### 3.2. End-of-Pipe Treatment Technology

The end-of-pipe treatment stage deals with the pollutants generated from former sub-stage to meet the national or industrial emission standards. In addition to the pollutants of the former stage, the input also includes the treatment investment of different pollutants, denoted as $X_{j}^{2p},p=1,2,3$. The outputs are the emissions of different pollutants [46,54], denoted as $G_{j}^{p},p=1,2,3$. The technology of end-of-pipe treatment ($T^{ET}$) is shown as Equation (4):

(4) $$\begin{array}{l} T^{ET}=\{(X^{2},Z,G)|\\ \sum_{j=1}^{n}\lambda_{j}^{2p}X_{j}^{2p}\leq X^{2p},p=1,2,3\\ \sum_{j=1}^{n}\lambda_{j}^{2p}Z_{pj}\geq Z_{p},p=1,2,3\\ \sum_{j=1}^{n}\lambda_{j}^{2p}G_{j}^{p}\leq G^{p},p=1,2,3\\ \lambda_{j}^{2p}\geq 0,p=1,2,3\} \end{array}$$

Similar, $\lambda_{j}^{2p}$ is the intensity vector of different end-of-pipe treatment stages. It should be noted that *Z*, as the generation of pollutants, is an undesirable input in the end-of-pipe treatment sub-stage [55], which means the more pollutants consumed in this stage, the better the environmental quality. Different from the previous pollution treatment technologies, the end-of-pipe treatment of industrial waste gas is further subdivided into the end-of-pipe treatment of SO_2_, NO_X_, and SD, expressed by P (*p* = 1,2,3).

### 3.3. Unified Technology of Industrial Waste Gas

Combining with the pollution prevention technology and the end-of-pipe treatment technology, the unified technology of industrial waste gas is given as follows:

(5) $$\begin{array}{l} T^{U}=\{(X,e,Y,Z,C,X^{2},G)|\\ \sum_{j=1}^{n}\lambda_{j}^{1}X_{i1j}\leq X_{i1},\;i1=1,\ldots ,m\\ a_{j}\sum_{j=1}^{n}\lambda_{j}^{1}e_{j}=e\\ \sum_{j=1}^{n}\lambda_{j}^{1}Y_{j}\geq Y\\ a_{j}\sum_{j=1}^{n}\lambda_{j}^{1}Z_{pj}=Z_{p},\;p=1,2,3\\ a_{j}\sum_{j=1}^{n}\lambda_{j}^{1}C_{j}=C\\ \sum_{j=1}^{n}\lambda_{j}^{2p}X_{j}^{2p}\leq X^{2p},p=1,2,3\\ \sum_{j=1}^{n}\lambda_{j}^{2p}Z_{pj}\geq Z_{p},p=1,2,3\\ \sum_{j=1}^{n}\lambda_{j}^{2p}G_{j}^{p}\leq G^{p},p=1,2,3\\ a_{j}\sum_{j=1}^{n}\lambda_{j}^{1}Z_{1j}\geq \sum_{j=1}^{n}\lambda_{j}^{21}Z_{1j}\\ a_{j}\sum_{j=1}^{n}\lambda_{j}^{1}Z_{2j}\geq \sum_{j=1}^{n}\lambda_{j}^{22}Z_{2j}\\ a_{j}\sum_{j=1}^{n}\lambda_{j}^{1}Z_{3j}\geq \sum_{j=1}^{n}\lambda_{j}^{23}Z_{3j}\\ \lambda_{j}^{1}\geq 0,\lambda_{j}^{2p}\geq 0\} \end{array}$$

As an intermediate output, *Z* is not only the output of the former sub-stage, but also the input of the latter sub-stage. The unified technology not only considers the output and input constraints of *Z*, but also constructs the connection constraints between two sub-stages through *Z*, that is, $a_{j}\sum_{j=1}^{n}\lambda_{j}^{1}Z_{pj}\geq \sum_{j=1}^{n}\lambda_{j}^{2p}Z_{pj},p=1,2,3$, which means that the optimal output of the former sub-stage should be greater than or equal to the optimal input of the latter sub-stage [56]. Since this paper mainly explores whether the generation and emission of industrial waste gas has the improvement potential, the following non-radial network DEA model is constructed:

(6) $$\begin{array}{l} E^{U}=\min\frac{1}{2}[\frac{1}{1+3}(\theta +\sum_{p=1}^{3}\varphi_{p})+\frac{1}{3}(\phi_{1}+\phi_{2}+\phi_{3})]\\ \sum_{j=1}^{n}\lambda_{j}^{1}X_{i1j}\leq X_{i10},\;i1=1,\ldots ,m\\ a_{j}\sum_{j=1}^{n}\lambda_{j}^{1}e_{j}=e_{0}\\ \sum_{j=1}^{n}\lambda_{j}^{1}Y_{j}\geq Y_{0}\\ a_{j}\sum_{j=1}^{n}\lambda_{j}^{1}Z_{pj}=\varphi_{p}Z_{p0},\;p=1,2,3\\ a_{j}\sum_{j=1}^{n}\lambda_{j}^{1}C_{j}=\theta C_{0}\\ \sum_{j=1}^{n}\lambda_{j}^{2p}X_{j}^{2p}\leq X_{0}^{2p},p=1,2,3\\ \sum_{j=1}^{n}\lambda_{j}^{2p}Z_{pj}\geq Z_{p0},p=1,2,3\\ \sum_{j=1}^{n}\lambda_{j}^{2p}G_{j}^{2p}\leq \phi_{p}G_{0}^{2p},p=1,2,3\\ a_{j}\sum_{j=1}^{n}\lambda_{j}^{1}Z_{1j}\geq \sum_{j=1}^{n}\lambda_{j}^{21}Z_{1j}\\ a_{j}\sum_{j=1}^{n}\lambda_{j}^{1}Z_{2j}\geq \sum_{j=1}^{n}\lambda_{j}^{22}Z_{2j}\\ a_{j}\sum_{j=1}^{n}\lambda_{j}^{1}Z_{3j}\geq \sum_{j=1}^{n}\lambda_{j}^{23}Z_{3j}\\ \sum_{j=1}^{n}\lambda_{j}^{1}=1,\sum_{j=1}^{n}\lambda_{j}^{2p}=1\\ 0\leq \theta ,\varphi_{t},\phi_{1},\phi_{2},\phi_{3}\leq 1\\ \lambda_{j}^{1}\geq 0,\lambda_{j}^{21}\geq 0,\lambda_{j}^{22}\geq 0,\lambda_{j}^{23}\geq 0 \end{array}$$

In Equation (6), different constraints are caused by different inputs and outputs. Specifically, in terms of the inputs and undesirable outputs, the less means the better, such as *X* and *G*. In contrary, the more desirable outputs (e.g., *Y*) are the better. In addition, the equality constraints of *e*, *Z*, and *C* embodies the cost disposability between energy and pollutants. The last three inequality constraints of *Z*, which demonstrate the link constraint between different sub-stages. Due to the different industrial development level among provinces, the model adopts the variable return on scale assumption, which is embodied by $\sum_{j=1}^{n}\lambda_{j}^{1}=1,\sum_{j=1}^{n}\lambda_{j}^{2p}=1$. Due to the existence of reduction ratio ($a_{j}$), the model is non-linear. Referring to Kuosman [57], letting $\mu_{j}^{1}=a_{j}\lambda_{j}^{1}$, $\varsigma_{j}^{1}=(1-a_{j})\lambda_{j}^{1}$, $\varsigma_{j}^{1}+\mu_{j}^{1}=\lambda_{j}^{1}$, Equation (6) is thus equivalent to Equation (7).

(7) $$\begin{array}{l} E^{U}=\min\frac{1}{2}[\frac{1}{1+3}(\theta +\sum_{p=1}^{3}\varphi_{p})+\frac{1}{3}(\phi_{1}+\phi_{2}+\phi_{3})]\\ \sum_{j=1}^{n}(\mu_{j}^{1}+\varsigma_{j}^{1})X_{i1j}\leq X_{i10},\;i1=1,\ldots ,m\\ \sum_{j=1}^{n}\mu_{j}^{1}e_{j}=e_{0}\\ \sum_{j=1}^{n}(\mu_{j}^{1}+\varsigma_{j}^{1})Y_{j}\geq Y_{0}\\ \sum_{j=1}^{n}\mu_{j}^{1}Z_{pj}=\varphi_{p}Z_{p0},\;p=1,2,3\\ \sum_{j=1}^{n}\mu_{j}^{1}C_{j}=\theta C_{0}\\ \sum_{j=1}^{n}\lambda_{j}^{2p}X_{j}^{2p}\leq X_{0}^{2p},p=1,2,3\\ \sum_{j=1}^{n}\lambda_{j}^{2p}Z_{pj}\geq Z_{p0},p=1,2,3\\ \sum_{j=1}^{n}\lambda_{j}^{2p}G_{j}^{2p}\leq \phi_{p}G_{0}^{2p},p=1,2,3\\ \sum_{j=1}^{n}\mu_{j}^{1}Z_{1j}\geq \sum_{j=1}^{n}\lambda_{j}^{21}Z_{1j}\\ \sum_{j=1}^{n}\mu_{j}^{1}Z_{2j}\geq \sum_{j=1}^{n}\lambda_{j}^{22}Z_{2j}\\ \sum_{j=1}^{n}\mu_{j}^{1}Z_{3j}\geq \sum_{j=1}^{n}\lambda_{j}^{23}Z_{3j}\\ \sum_{j=1}^{n}\left(\mu_{j}^{1}+\varsigma_{j}^{1}\right)=1,\sum_{j=1}^{n}\lambda_{j}^{2p}=1\\ 0\leq \theta ,\varphi_{t},\phi_{1},\phi_{2},\phi_{3}\leq 1\\ \mu_{j}^{1}\geq 0,\varsigma_{j}^{1}\geq 0,\lambda_{j}^{2p}\geq 0 \end{array}$$

It can be seen that the unified efficiency includes two parts, $(\theta +\sum_{p=1}^{3}\varphi_{p})/(1+3)$ and $(\phi_{1}+\phi_{2}+\phi_{3})/3$, which correspond to the reduction potential of generation and emission of pollutants, respectively. We define the above two parts as the efficiency of pollution prevention and end-of-pipe treatment, expressed as $E^{PP}$ and $E^{ET}$. If and only if $E^{PP}=E^{ET}=1$, the unified efficiency is effective, otherwise, it indicates that DMU has room for improvement. Our model can further obtain end-of-pipe treatment efficiencies of three air pollutants to explore the internal inefficiencies of this sub-stage.

The above non-radial network DEA model can accurately measure the pollutant prevention efficiency and end-of-pipe treatment efficiency of DMU at a certain time. However, the efficiency of different periods is measured by different frontier, which is not comparable [58]. Here, the global technology, proposed by Oh [59], is introduced to explore inter-temporal efficiency change. The global technology includes DMUs of all periods so that the responding frontier are common and the efficiencies of different periods are comparable.

Suppose that there are T study periods in total and the unified efficiency of period i can be calculated by following global non-radial network DEA model:

(8) $$\begin{array}{l} E^{GU}=\min\frac{1}{2}[\frac{1}{1+3}(\theta^{i}+\sum_{p=1}^{3}\varphi_{p}^{i})+\frac{1}{3}(\phi_{1}^{i}+\phi_{2}^{i}+\phi_{3}^{i})]\\ \sum_{t=1}^{T}\sum_{j=1}^{n}(\mu_{j}^{1t}+\varsigma_{j}^{1t})X_{i1j}^{t}\leq X_{i10}^{i},\;i1=1,\ldots ,m\\ \sum_{t=1}^{T}\sum_{j=1}^{n}\mu_{j}^{1t}e_{j}^{t}=e_{0}^{i}\\ \sum_{t=1}^{T}\sum_{j=1}^{n}(\mu_{j}^{1t}+\varsigma_{j}^{1t})Y_{j}^{t}\geq Y_{0}^{i}\\ \sum_{t=1}^{T}\sum_{j=1}^{n}\mu_{j}^{1t}Z_{pj}^{t}=\varphi_{p}^{i}Z_{p0}^{i},\;p=1,2,3\\ \sum_{t=1}^{T}\sum_{j=1}^{n}\mu_{j}^{1t}C_{j}^{t}=\theta^{i}C_{0}^{i}\\ \sum_{t=1}^{T}\sum_{j=1}^{n}\lambda_{j}^{2pt}X_{j}^{2pt}\leq X_{0}^{2pi},p=1,2,3\\ \sum_{t=1}^{T}\sum_{j=1}^{n}\lambda_{j}^{2pt}Z_{pj}^{t}\geq Z_{p0}^{i},p=1,2,3\\ \sum_{t=1}^{T}\sum_{j=1}^{n}\lambda_{j}^{2pt}G_{j}^{2pt}\leq \phi_{p}^{i}G_{0}^{2pi},p=1,2,3\\ \sum_{t=1}^{T}\sum_{j=1}^{n}\mu_{j}^{1t}Z_{1j}^{t}\geq \sum_{t=1}^{T}\sum_{j=1}^{n}\lambda_{j}^{21}Z_{1j}^{t}\\ \sum_{t=1}^{T}\sum_{j=1}^{n}\mu_{j}^{1t}Z_{2j}^{t}\geq \sum_{t=1}^{T}\sum_{j=1}^{n}\lambda_{j}^{22}Z_{2j}^{t}\\ \sum_{t=1}^{T}\sum_{j=1}^{n}\mu_{j}^{1t}Z_{3j}^{t}\geq \sum_{t=1}^{T}\sum_{j=1}^{n}\lambda_{j}^{23}Z_{3j}^{t}\\ \sum_{t=1}^{T}\sum_{j=1}^{n}\left(\mu_{j}^{1t}+\varsigma_{j}^{1t}\right)=1,\sum_{t=1}^{T}\sum_{j=1}^{n}\lambda_{j}^{2pt}=1\\ 0\leq \theta^{i},\varphi_{h}^{i},\phi_{p}^{i}\leq 1\\ \mu_{j}^{1t}\geq 0,\varsigma_{j}^{1t}\geq 0,\lambda_{j}^{2pt}\geq 0,p=1,2,3,\;t=1,\ldots ,T \end{array}$$

The efficiencies of pollution prevention and end-of-pipe treatment in global technology can be obtained with the similar way as previous section. If and only if the efficiency of pollution prevention and end-of-pipe treatment of all periods are effective, the DMU is effective.

Compared with the existing network DEA model, our model has the following advantages: (1) Considering the pollution prevention and end-of-pipe treatment of pollutants for the first time, it is more in line with China’s industrial development; (2) Constructing a global non-radial network DEA model, focusing on whether there is room for improvement in the generation and emission of industrial waste gas in China, which is more targeted; (3) Dividing end-of-pipe treatment into three parallel sub-stages, corresponding to SO_2_, NO_X_, and SD, respectively, allowing to further explore the internal inefficiency of this stage.

### 3.4. Grey Relation Analysis

In this part, the GRA is adopted to figure out the key factor which affects the unified efficiency to help decision maker to maximize the utility of limited sources. The specific steps of GRA are as follows:

1. Defining $Y_{k},(k=1,\ldots ,n)$ and $X_{ik},(i=1,\ldots m;k=1,\ldots ,n)$ as reference sequence and comparability sequence, which refer to the unified efficiency of our global non-radial network DEA model and generation and emission amount of air pollutants, respectively. In this paper, *n* (*n* = 30) indicates the number of DMUs and *m* (*m* = 7) denotes the type of the generation and emission of air pollutants.
2. Standardization of the reference sequence and comparability sequence, expressed by $Y_{k}^{\prime },(k=1,\ldots ,n)$ and $X_{ik}^{\prime },(i=1,\ldots m;k=1,\ldots ,n)$. The new sequences are given as follows:
  (9) $$\Delta_{ik}=\left|Y_{k}^{\prime }-X_{ik}^{\prime }\right|,(i=1,\ldots m;k=1,\ldots ,n)$$
3. Relation coefficients of different sequences can be calculated by following formula:
  (10) $$\xi_{ik}=\frac{\underset{i}{\min}\;\underset{k}{\min}\;\Delta_{ik}+\rho \;\underset{i}{\max}\;\underset{k}{\max}\;\Delta_{ik}}{\Delta_{ik}+\rho \;\underset{i}{\max}\;\underset{k}{\max}\;\Delta_{ik}},(i=1,\ldots m;k=1,\ldots ,n)$$
  where ρ∈(0,∞) means the distinguishing coefficient and the smaller ρ, the better the discrimination. Referring to Xu et al. [60], we set ρ=0.5.
4. Calculation of relation degree:
  (11) $$\gamma_{i}=\sum_{k}^{n}\xi_{ik},i=1,\ldots ,m$$
  where $\gamma_{i}\in (1,0)$ indicates the relation degree of comparability sequence *i*. We can rank different factors according to $\gamma_{i}$. The bigger $\gamma_{i}$, the greater the importance, which means that limited resources should be given priority with the factor.

### 3.5. Indicator Select and Data Source

By applying the proposed global non-radial network DEA model, we collect the panel data of 30 provincial industrial systems from 2011 to 2015 to explore the unified efficiency of industrial waste gas considering the pollution prevention and end-of-pipe treatment (Tibet, Hong Kong, Macao, and Taiwan are excluded due to data unavailability). The specific indexes are shown in Figure 2. In pollution prevention stage, the industrial energy consumption, total assets and the average number of employees of industrial enterprises above the designated scale are used to produce economic output (industrial value-added), accompanied with generation of SO_2_, NO_X_, and SD. In addition, as the main component of greenhouse gas [61], CO_2_ is another pollutant cannot be ignored. In end-of-pipe treatment stage, expenditures for desulfurization, denitrification and dedust are additional inputs except the generation of pollution from former stage. With regard to outputs, we choose the pollution emission of SO_2_, NO_X_, and SD. Since there is no official data for industrial CO_2_ emission, we firstly compute provincial CO_2_ emission following previous research [11] which includes seven main fuels. Then, industrial CO_2_ emission can be obtained according to following formula:

(12) $${\mathrm{CO}}_{2}^{in}=\frac{industiral\;value-added}{\mathrm{GDP}}\times {\mathrm{CO}}_{2}^{pr}$$

The calculation of provincial CO_2_ emission can be estimated using following formula:

(13) $${\mathrm{CO}}_{2}^{pr}=\sum_{h=1}^{7}{EC}_{h}\times {CEC}_{h}=\sum_{h=1}^{7}{EC}_{h}\times {CC}_{h}\times H_{h}\times O_{h}\times (44/12)$$

where ${EC}_{h}$ represents provincial consumption of fossil fuel h. ${CEC}_{h}$ indicates the carbon emission coefficient of fossil fuel h, which includes ${CC}_{h}$,$H_{h}$,$O_{h}$ and (44/12), representing the carbon content, the heat equivalent, carbon oxidation factor of fossil fuel h and the molecular weight ratio of CO_2_ to C, respectively. The corresponding coefficient value is shown in Table 1.

All these data are collected from the China Statistical Yearbook (2012–2016), China Industrial Statistic Yearbook (2012–2016), China Environment Statistic Yearbook (2012–2016), China Energy Statistical Yearbook (2012–2016), and statistic yearbook of individual province (2012–2016). Individual missing data is obtained by interpolation. Table 2 lists the descriptive statistics of these indexes.

## 4. Empirical Analysis

### 4.1. Analysis of the Unified and Sub-Stage Efficiencies

The unified and sub-stage efficiencies of China’s industrial waste gas are calculated with the proposed global non-radial network DEA model. The specific efficiency is listed in Table 3.

Firstly, for the unified efficiency, none of the provinces is always efficient during the sample period. Only Beijing in 2015, Shandong in 2014, and Hainan in 2013 and 2015 can be considered efficient, with a unified efficiency of 1. The top three provinces are Hainan, Shandong, and Inner Mongolia. The last three provinces include Shanxi, Shaanxi, and Zhejiang. The overall average efficiency of China’s industrial waste gas is 0.725 which has a large room for improvement and more than a third of provinces fail to reach this level. Three of these provinces (Beijing, Liaoning, and Zhejiang) are located in the eastern area, six (Shanxi, Jilin, Anhui, Jiangxi, Hubei, and Heilongjiang) in the central area, and three (Shaanxi, Chongqing, and Ningxia) in the western area (The specific area division is put in the next section). From a development perspective, the average unified efficiency shows an obvious downward trend form 0.766 in 2011 to 0.692 in 2014 and a rapid rise in 2015. The possible explains for this increase in efficiency was that the Chinese government issued the action plan for the prevention and control of air pollution in September 2013, but the effect of policy occurred a certain delay.

**Figure 4:**
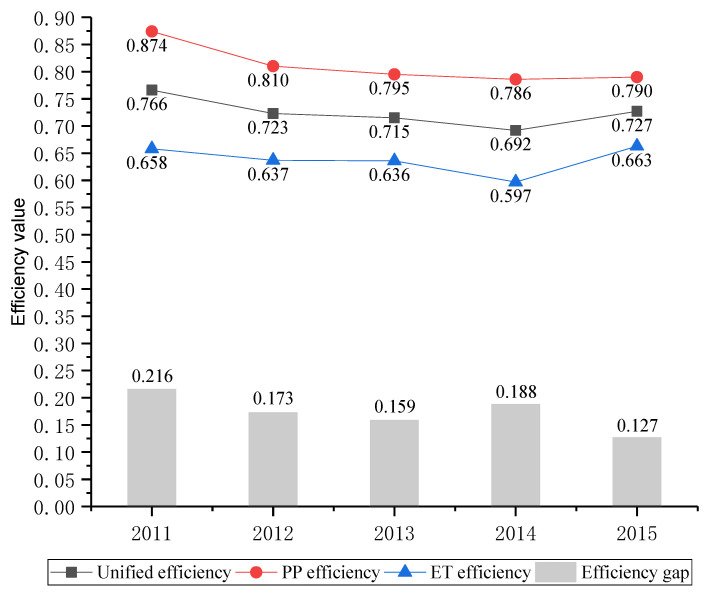
The annual efficiency change and gap.

Secondly, when focusing on pollution prevention and end-of-pipe treatment efficiencies, great differences can be found. Compared with ET stage, the efficiency of PP stage is relatively high and the number of efficient DMUs is quite large. Specifically, the PP efficiencies of nine provinces are always efficient in sample period, and other 14 provinces are efficient in some years. However, there are only three provinces whose ET efficiencies are efficient in individual years. It can be inferred that the inefficiency in the ET stage is the major cause to the inefficiency of the unified efficiency. From the development perspective, as shown in Figure 4, the average PP efficiency and ET efficiency have a similar development trend with the average unified efficiency. And the ET efficiency has a bigger augment than PP in 2015.

It can be seen from Table 4 that the efficiency gap between PP and ET stage has gradually narrowed from the 0.216 in 2011 to 0.127 in 2015, showing an improving trend. And the minimum efficiency gap is only 0.127 in 2015.

**Figure 5:**
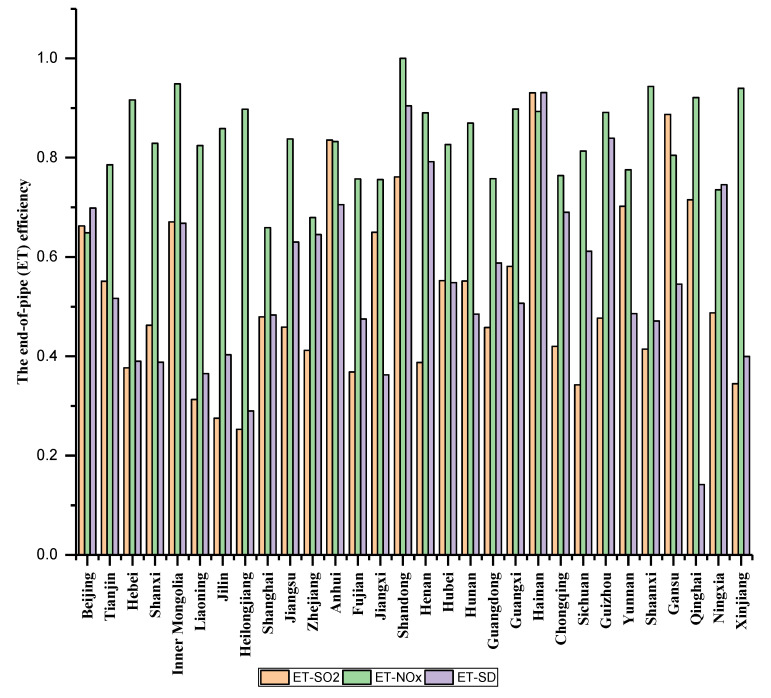
Average ET efficiency of SO_2_, NOx, and soot and dust.

To further figure out the cause of low efficiency in ET stage, Figure 5 gives the average ET efficiencies of SO_2_, NO_X_, and SD, which are denoted as ET-SO_2_, ET-NO_X_, and ET-SD, respectively. In Figure 5, it can be seen that the ET efficiency of NO_X_ performs well in most provinces. In contrast, the ET efficiencies of SO_2_ and soot and dust have different characteristics in different provinces. For example, provinces including Tianjin, Shanxi, Anhui, Jiangxi, and so on, have a high ET efficiency of SO_2_. Provinces such as Hebei, Jilin, Shandong, and Henan have a high ET efficiency of soot and dust. In other words, the weak-links of ET stage in different provinces are different, which requires them to formulate targeted policies to improve the end-of-pipe treatment efficiency.

For the purpose of verifying whether the ET efficiencies of SO_2_, NO_X_, and SD are significantly different, Wilcoxon-Mann-Whitney test is used to test the hypothesis that there is no difference in any two group efficiencies. The results of the test are presented in Table 4. We can find a significant difference in the ET efficiency between two groups, that is, ET-SO_2_ and ET-NO_X_, and ET-NO_X_ and ET-SD. That means it is essential to separately measure the ET efficiency of different pollutants.

**Figure 6:**
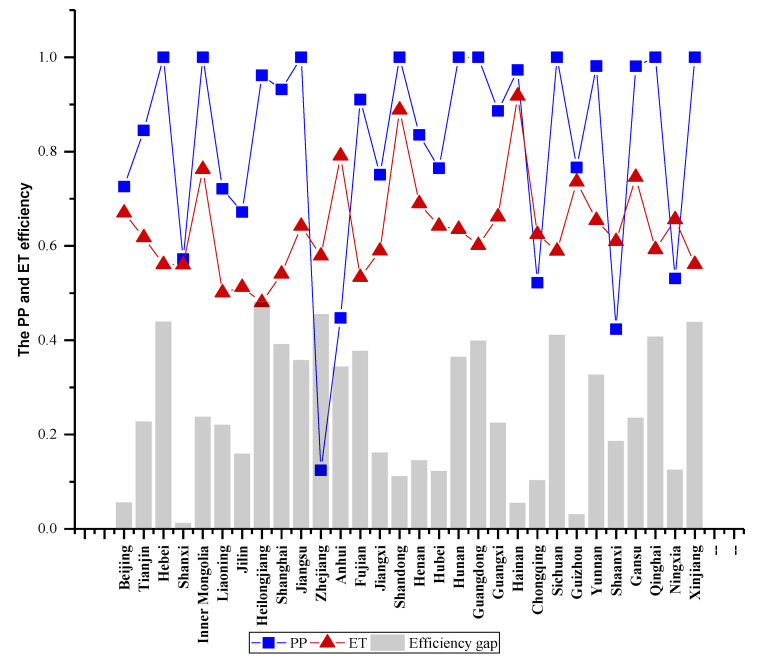
Average efficiency and gap of PP and ET stage.

Thirdly, although the average PP efficiency is superior to that of ET in most provinces, some provinces have the opposite situation. Figure 6 shows the average PP and ET efficiencies of 30 provinces. We can find that most provinces have a larger PP efficiency except Zhejiang, Anhui, Chongqing, Shaanxi, and Ningxia, which means it is necessary for different provinces to make different improvement direction. Another phenomenon to be pointed out is that there are big efficiency gaps between PP and ET stage in many provinces. Among them, Heilongjiang has the biggest efficiency gap, reaching 0.482. That indicates that reducing the efficiency gaps between the two stages is also the significant task for many provinces.

Finally, Table 3 also shows that the unified efficiency is efficient when both the PP efficiency and ET efficiency are efficient at the same time. Accordingly, only Beijing in 2015, Shandong in 2014, and Hainan in 2013 and 2015 perform well in the sample period. Most provinces have the potential to further reduce the generation and emission of air pollutants from the perspective of pollution prevention and end-of-pipe treatment.

### 4.2. Area Efficiency Analysis

Due to the fluidity characteristics of air pollutants, the air quality of a province may be affected by the neighboring provinces. To realize the joint prevention and control of air pollutants among areas, it is necessary to figure out the deficiency of different areas. Referring to previous research [62,63], 30 provinces are geographically grouped into three areas: the eastern area, the central area, and the western area. The specific area divisions are listed in Table 5.

**Figure 7:**
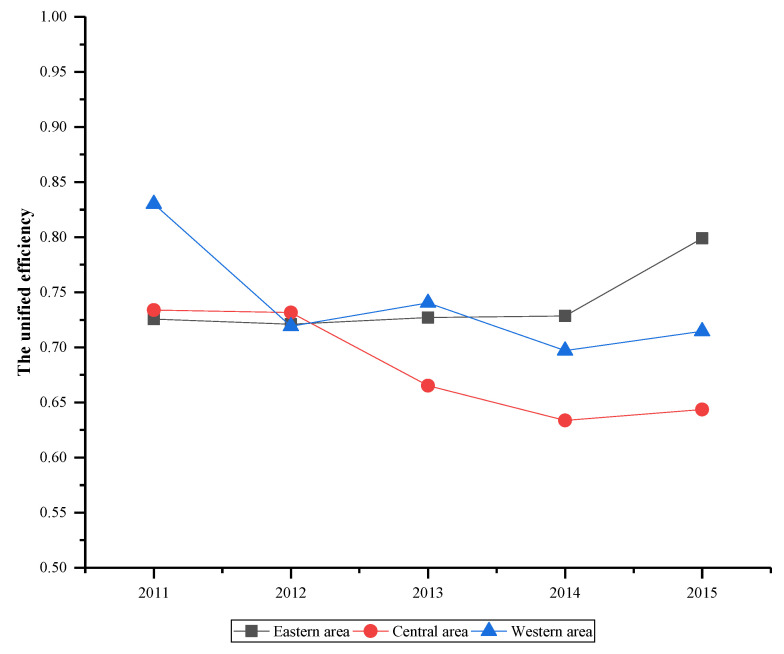
The unified efficiency of three areas.

Figure 7 demonstrates the unified efficiency of China’s industrial waste gas in three areas. It can be seen that the unified efficiency of three areas shows different change trends and these changes are also different from the unified efficiency in Figure 4. However, the efficiency of three areas increased between 2014 and 2015, which was the same as the unified efficiency change. Overall, the performance of the eastern area is stable and shows an optimistic development. While efficiencies of central and western areas are relatively poor, especially for central area from 2012 to 2014, which has a significant decline. After 2012, the efficiency differences among areas initiate expansion, indicating the area disparity is gradually widening, which should arouse the alarm of relevant departments.

**Figure 8:**
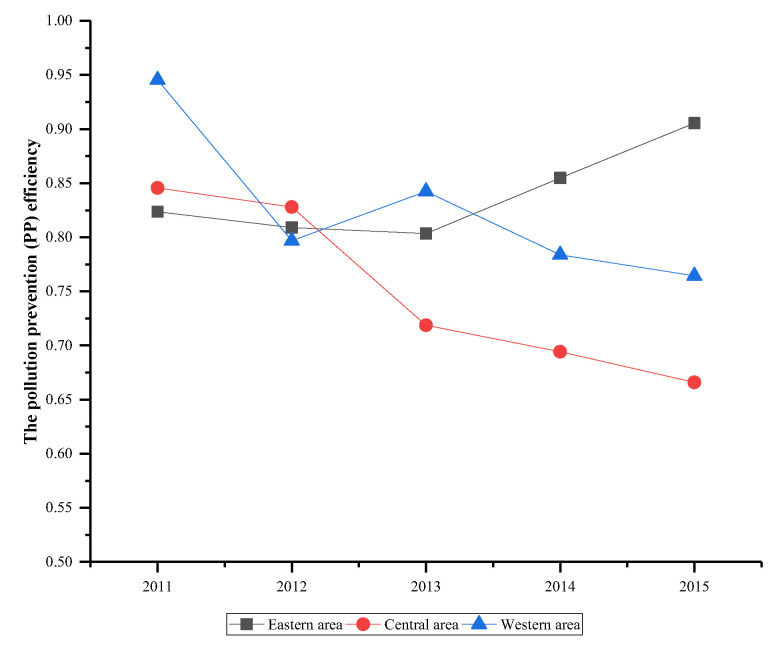
The average PP efficiency of three areas.

**Figure 9:**
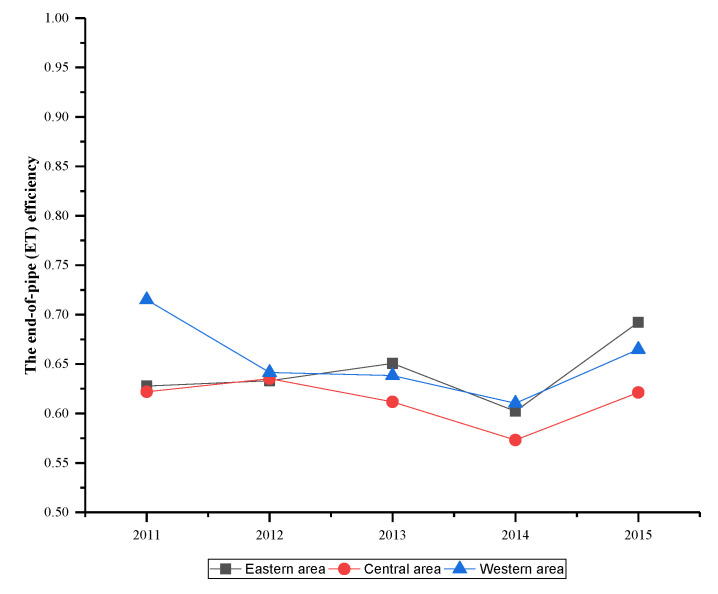
The average ET efficiency of three areas.

Figure 8 and Figure 9 illustrate the average PP and ET efficiency of three area from 2011 to 2015. Compared with PP efficiency, the ET efficiency of three areas has a similar change with the unified efficiency shown in Figure 7. Combining with PP and ET efficiencies of three areas between 2014 and 2015, we can find the driving factor of the unified efficiency improvement are different in three areas. For the eastern area, the unified efficiency improvement was attributed to the joint effect of PP and ET efficiency. But for central and western areas, the unified efficiency increase is mainly driven by the ET efficiency. Although ET efficiency of central and western areas performs well between 2014 and 2015, the PP efficiency is still higher than ET efficiency. In order to improve the air quality in the central and western areas, the government should not only pay attention to the low efficiency of EE even if it performs well, but also attach importance to the decline of PP efficiency.

**Figure 10:**
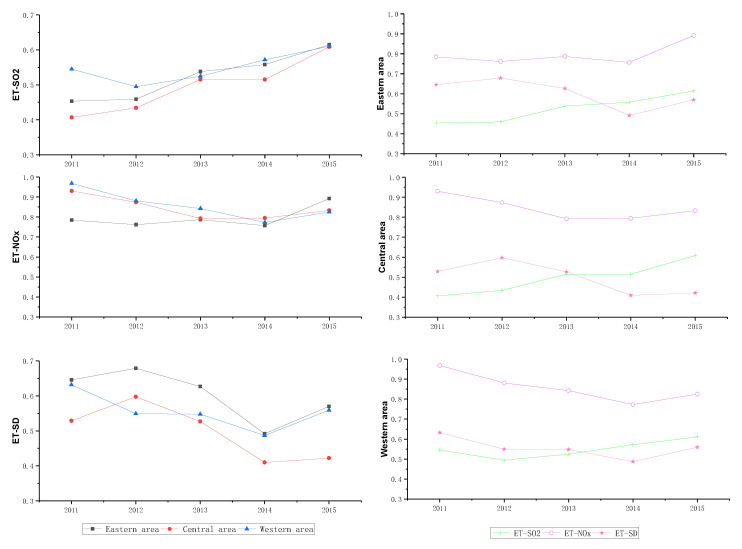
The ET efficiency of SO_2_, NOx, and SD.

Additionally, the ET efficiency of SO_2_, NO_X_, and SD in three areas can be obtained and the results are shown in Figure 10, which are demonstrated from different areas and pollutants, respectively. As can be seen from the right three images of Figure 10, no matter which area is concerned, the ET efficiency of NO_X_ is the highest among three pollutants. Specifically, for ET-NO_X_, the efficiency of eastern area shows a wave-like upward trend, while the efficiency of central and western areas shows a trend of decreasing first and then rising and the efficiency in 2015 is less than that in 2011. For ET-SO_2_ and ET-SD, there are clear efficiency differences in three areas, which can be seen from the left three images of Figure 10. That is, the ET efficiency of SO_2_ in all areas increases gradually in the sample period. However, the ET efficiency of SD in three areas is irregular and all areas have a lower efficiency in 2015 than initial year. So, from the perspective of three air pollutants, more attention should be paid to the governance deficiency of SD.

It is worth noting that for different areas, the weak links are also different. As shown in Figure 10, comparing with the eastern and western areas, bigger differences exist in the ET efficiency of three pollutants in the central area, which means both efficiency improvement and efficiency gap reduction should be strengthened for the central area.

### 4.3. Improvement Direction Analysis

To maximize the effect of limited resources, GRA is used to figure out the key factor affecting the unified efficiency from four pollutants (the generation and emission of SO_2_, NOx, and SD, and the emission of CO_2_). As shown in Table 6, each pollutant has a great impact on the unified efficiency, and the values of GRA are more than 0.6 for both the national and different areas, which is reasonable since the unified efficiency is obtained from the generation and emission reduction potential of pollutants. However, for different pollutants and areas, there are also some specific distinctions. From the perspective of generation and emission of pollutants, the emissions of SO_2_, NO_X_, and SD have a bigger impact on the unified efficiency in the national and central area, which is shown in bold font. But the opposite is true in the eastern area. That is, the generation of SO_2_, NO_X_, and soot and dust is more important for the unified efficiency improvement in eastern area. For the western area, the generation of SD and the emission of SO_2_ and NO_X_ is more influential. Above observations indicate that the improvement direction of air pollution should be different in each area.

From the perspective of pollutants, the influence on the unified efficiency is also diverse. For example, the pollutant that have the greatest influence on the unified efficiency in the eastern area is the generation of NO_X_. For central area, the pollutant is the emission of SD. However, the generation of SD should be given more priority in western area.

### 4.4. Comparative Analysis

**Figure 11:**
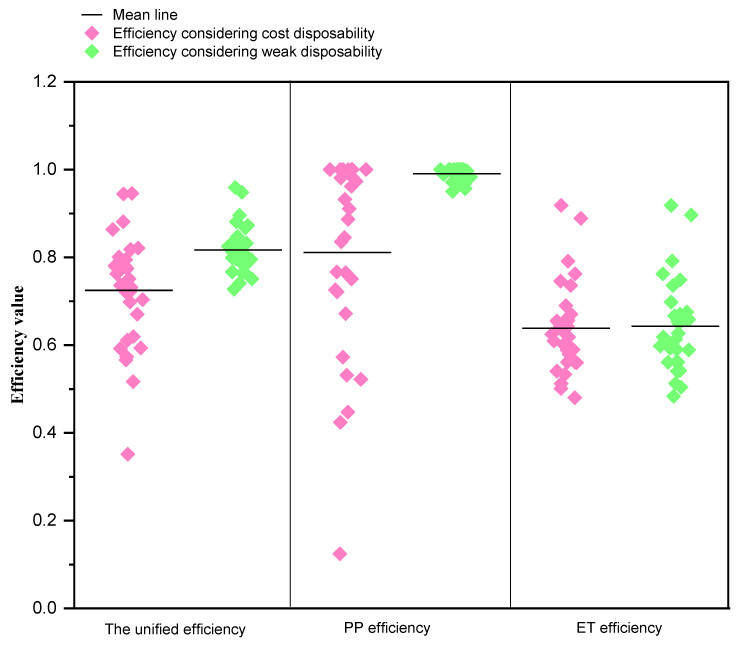
The efficiency comparison of different models.

Section 4.1 verified the necessity of distinguishing different pollutants in the ET stage. To show the superiority of our model, Figure 11 further presents the efficiency comparison between our model and the model considering weak disposability which is popular in industrial efficiency evaluation. The detailed model is presented in Appendix A.

As can be seen from Figure 11, ignoring the potential relationship between energy and pollutants lead to the overestimate of efficiencies both the unified and PP efficiency, which is shown by the different mean lines. And the number of efficient DMUs increase significantly, especially for the PP stage, which greatly reduces the discrimination power of model. However, there are no big differences in ET efficiency between two models, which is rational since pollution prevention occurs in the production sub-stage. In short, our model has more advantages both at practical level and at model result.

## 5. Conclusions

As the important ways to reduce environmental pressure and achieve the goal of energy conservation and emission reduction, pollution prevention and end-of-pipe treatment are common in China current industrial system. In this paper, a global non-radial network DEA model, combining with pollution prevention and end-of-pipe treatment, was proposed to explore the potential reduction of generation and emission of air pollutants including SO_2_, NO_X_, SD, and CO_2_. Then, GRA is used to figure out the key factor affecting the unified efficiency. To our best of knowledge, this is the first attempt to study industrial air pollutants from the perspective of integration of PP efficiency and ET efficiency.

The following findings were obtained: (1) the average unified efficiency of China’s industrial waste gas is only 0.725, which offers a large room for improvement and most provinces have the potential to further reduce the generation and emission of air pollutants. (2) compared with the efficiency of pollution prevention, the low efficiency of end-of-pipe treatment is the major contributor to the inefficiency of the unified efficiency in most provinces and the efficiency differences between PP and ET is gradually shrinking. (3) the unified efficiency in eastern area performs well which is the joint effect of PP and ET stage, especially in 2014–2015, but for the central and western areas, the unified efficiency increase is mainly driven by the ET efficiency. (4) after 2012, significant area disparities can be found and PP efficiencies in the central and western areas have an obvious decline. (5) the ET efficiency of NO_X_ is higher than SO_2_ and SD. In contrast, the treatment of SD should be given greater priority.

From the perspective of pollution prevention and end-of-pipe treatment, following policies are proposed. Most provinces have a higher PP efficiency compared with ET efficiency. Thus, the improvement of ET efficiency should be given great priority. On the one hand, the research and development (R&D) and upgrading of advanced technologies in desulfurization, denitrification, and dedust are urgent for the improvement of end-of-pipe treatment efficiency of different air pollutants, which requires more financial support from central and local governments. On the other hand, the sharing of treatment technology among different areas should be strengthened. For Zhejiang, Anhui, Chongqing, Shaanxi, and Ningxia who have a bigger ET efficiency, the measures such as the development of clean energy and the adjustment of industrial structure are momentous. In addition, there are great differences between PP and ET efficiencies for many provinces. Therefore, the coordination mechanism of PP stage and ET stage is also indispensable for improving the unified efficiency.

From the perspective of pollutants, the ET efficiency of NOx performs well for both national and different areas. In this way, more attention should be put the treatment of SO_2_ and SD. It is necessary for local governments to formulate effective pollution monitoring mechanism according to their weak links. Then, different tax relief policies are helpful for the emission reduction of different pollutants. Specifically, for the industry with low treatment efficiency of pollutants, the local government should give more stricter tax policy. Improving the environmental protection awareness of people and establishing convenient tip-off channels can also contribute to the improvement of the unified efficiency.

From the perspective of areas, except the ET inefficiency, the decline of PP efficiency should not be ignored in the central and western areas. That requires them to speed up the pace of industrial restructuring and pay more attention to the role of pollution prevention. The results of GRA also indicate differentiated policies should be constructed for different areas. That is, the generation of NO_X_, the emission of SD, and the generation of SD should be given great preference for the eastern, central, and western areas, respectively.

This study mainly focused on exploring the reduction potential of generations and emissions of air pollutants from the perspective of pollution prevention and end-of-pipe treatment. Although the necessity and superiority of our model have been emphasized, there may be some potential limitations. For example, the indicators are treated as precise in our model, while the uncertainty of data is common in reality. The proposed model mainly considered the generations and emissions of pollutants. It is interesting to comprehensively evaluate the inefficiency of all indicators, which can also be a research indirection in future.

## Figures and Tables

**Table 1:** Coefficients of carbon emissions of different energy.

Fuels	Coal	Coke	Kerosene	Petrol	Diesel	Fuel Oil	Natural Gas
CC	27.28	29.41	19.60	18.90	20.17	21.09	15.32
H	178.24	284.35	447.50	448.00	433.30	401.90	0.38
O (%)	92.30	92.80	98.60	98.00	98.20	98.50	99.00

CC, H, and O indicate the carbon content, the heat equivalent, carbon oxidation factor of energy.

**Table 2:** Statistical description of index.

Indexes	Obs	Min	Max	Mean	Std.Dev
The employees of industrial sector	150	11.64	2338.38	394.13	437.60
Industrial total investment	150	1747.91	107,061.73	28,614.49	23,106.27
Industrial energy consumption	150	826.98	30,070.00	9308.00	6631.21
Industrial value-added	150	475.04	30,259.49	8676.68	6956.00
Industrial CO_2_	150	369.31	40,986.40	13,341.12	10,341.26
The generation of industrial SO_2_	150	64,153.00	7,075,696.00	2,069,282.15	1,368,603.70
The generation of industrial NOX	150	52,233.00	1,579,379.00	620,930.57	380,343.17
The generation of industrial SD	150	1,461,648.00	71,873,762.00	25,910,672.45	16,713,897.94
The expenditure of desulfurization	150	9017.70	795,303.90	201,319.42	163,376.35
The expenditure of denitrification	150	0.61	296,439.30	52,691.54	59,984.36
The expenditure of dedust	150	10,399.70	1,612,885.60	226,851.46	213,070.39
The emission of industrial SO_2_	150	22,070.00	1,628,647.00	604,034.45	357,806.76
The emission of industrial NO_X_	150	26,864.00	1,273,603.00	501,136.73	312,733.93
The emission of industrial SD	150	10,660.00	1,450,723.00	394,071.07	280,684.17

Obs and Std.Dev are the abbreviation of absolute value and standard deviation, respectively.

**Table 3:** The efficiency of industrial waste gas.

Provinces/Years	$E^{U}$					$E^{PP}$					$E^{ET}$				
	2011	2012	2013	2014	2015	2011	2012	2013	2014	2015	2011	2012	2013	2014	2015
Beijing	0.635	0.620	0.598	0.637	1.000	0.740	0.696	0.584	0.610	1.000	0.531	0.544	0.612	0.665	1.000
Tianjin	0.691	0.709	0.727	0.780	0.750	0.782	0.761	0.682	1.000	1.000	0.600	0.657	0.771	0.561	0.501
Hebei	0.794	0.802	0.763	0.747	0.796	1.000	1.000	1.000	1.000	1.000	0.588	0.604	0.526	0.494	0.592
Shanxi	0.554	0.544	0.551	0.579	0.603	0.524	0.520	0.595	0.618	0.605	0.584	0.568	0.507	0.541	0.601
Inner Mongolia	0.935	0.855	0.852	0.853	0.912	1.000	1.000	1.000	1.000	1.000	0.869	0.710	0.704	0.706	0.823
Liaoning	0.618	0.612	0.593	0.584	0.648	0.647	0.701	0.683	0.753	0.823	0.589	0.524	0.502	0.416	0.474
Jilin	0.704	0.641	0.609	0.537	0.469	0.882	0.718	0.710	0.577	0.470	0.527	0.563	0.507	0.496	0.468
Heilongjiang	0.758	0.753	0.742	0.678	0.675	1.000	1.000	1.000	0.916	0.893	0.515	0.506	0.484	0.439	0.457
Shanghai	0.708	0.713	0.734	0.713	0.812	0.877	0.860	0.923	1.000	1.000	0.539	0.566	0.546	0.427	0.625
Jiangsu	0.799	0.848	0.811	0.799	0.847	1.000	1.000	1.000	1.000	1.000	0.599	0.696	0.622	0.599	0.695
Zhejiang	0.332	0.373	0.350	0.339	0.364	0.106	0.114	0.129	0.134	0.137	0.558	0.631	0.571	0.544	0.590
Anhui	0.631	0.621	0.621	0.605	0.617	0.505	0.500	0.408	0.426	0.398	0.758	0.742	0.835	0.784	0.837
Fujian	0.704	0.706	0.709	0.728	0.763	0.907	0.901	0.838	0.906	1.000	0.501	0.510	0.580	0.550	0.526
Jiangxi	0.802	0.804	0.601	0.583	0.562	1.000	1.000	0.618	0.599	0.538	0.603	0.607	0.585	0.566	0.586
Shandong	0.922	0.899	0.921	1.000	0.979	1.000	1.000	1.000	1.000	1.000	0.843	0.798	0.843	1.000	0.959
Henan	0.882	0.877	0.706	0.654	0.695	1.000	1.000	0.744	0.732	0.701	0.763	0.754	0.668	0.577	0.689
Hubei	0.753	0.778	0.676	0.634	0.678	0.855	0.886	0.675	0.686	0.722	0.651	0.669	0.678	0.581	0.634
Hunan	0.787	0.836	0.815	0.800	0.849	1.000	1.000	1.000	1.000	1.000	0.575	0.673	0.630	0.601	0.699
Guangdong	0.798	0.777	0.792	0.809	0.827	1.000	1.000	1.000	1.000	1.000	0.597	0.553	0.584	0.618	0.654
Guangxi	0.870	0.704	0.738	0.743	0.816	1.000	0.713	0.826	0.893	1.000	0.740	0.694	0.650	0.593	0.632
Hainan	0.980	0.873	1.000	0.876	1.000	1.000	0.865	1.000	1.000	1.000	0.961	0.880	1.000	0.751	1.000
Chongqing	0.869	0.515	0.516	0.464	0.502	1.000	0.445	0.408	0.358	0.399	0.739	0.585	0.624	0.570	0.606
Sichuan	0.844	0.854	0.798	0.749	0.728	1.000	1.000	1.000	1.000	1.000	0.689	0.708	0.596	0.498	0.455
Guizhou	0.901	0.827	0.690	0.710	0.628	1.000	1.000	0.658	0.700	0.474	0.801	0.653	0.722	0.720	0.783
Yunnan	0.850	0.797	0.734	0.837	0.873	1.000	1.000	0.907	1.000	1.000	0.700	0.593	0.560	0.673	0.747
Shaanxi	0.504	0.497	0.540	0.503	0.539	0.399	0.415	0.468	0.411	0.425	0.609	0.579	0.613	0.595	0.652
Gansu	0.905	0.893	0.865	0.784	0.869	1.000	1.000	1.000	0.905	1.000	0.810	0.787	0.730	0.663	0.739
Qinghai	0.856	0.765	0.815	0.800	0.746	1.000	1.000	1.000	1.000	1.000	0.711	0.529	0.630	0.600	0.493
Ningxia	0.841	0.443	0.844	0.485	0.354	1.000	0.191	1.000	0.353	0.111	0.682	0.696	0.689	0.617	0.597
Xinjiang	0.757	0.761	0.752	0.740	0.893	1.000	1.000	1.000	1.000	1.000	0.515	0.523	0.504	0.480	0.785
Average	0.766	0.723	0.715	0.692	0.727	0.874	0.810	0.795	0.786	0.790	0.658	0.637	0.636	0.597	0.663

$E^{U}$, $E^{PP}$, and $E^{ET}$ respectively indicate the unified efficiency, pollution prevention efficiency and end-of-pipe treatment efficiency.

**Table 4:** Wilcoxon-Mann-Whitney test.

ET Efficiencies	ET-SO_2_	ET-NOx	ET-SD
ET-SO_2_	-	0.000 ***	0.391
ET-NOx	0.000 ***	-	0.000 ***

*** < 0.01.

**Table 5:** Area division in China.

Areas	Provinces
The eastern area (11 provinces)	Beijing, Tianjin, Hebei, Liaoning, Shandong, Shanghai, Jiangsu, Zhejiang, Fujian, Guangdong, Hainan
The central area (8 provinces)	Shanxi, Jilin, Heilongjiang, Anhui, Jiangxi, Henan, Hubei, Hunan
The western area (11 provinces)	Chongqing, Sichuan, Guizhou, Yunnan, Shaanxi, Gansu, Qinghai, Ningxia, Xinjiang, Inner Mongolia, Guangxi

**Table 6:** The value of GRA.

Pollutants/Areas	National	The Eastern Area	The Central Area	The Western Area
PP-SO_2_	0.750	**0.686**	0.665	0.769
PP-NO_X_	0.732	**0.705**	0.656	0.763
PP-SD	0.743	**0.683**	0.623	**0.796**
PP-CO_2_	0.707	0.688	0.650	0.750
ET-SO_2_	**0.762**	0.682	**0.672**	**0.790**
ET-NO_X_	**0.740**	0.702	**0.663**	**0.778**
ET-SD	**0.745**	0.680	**0.704**	0.786

The bold font shows a higher value between PP and ET for the same pollutant.

## Abbreviations

DEA	Data envelopment analysis	*X*	Neutral inputs of the PP stage
PP	Pollution prevention	*e*	Energy input of the PP stage
ET	End-of-pipe treatment	*Y*	Desirable output of the PP stage
SD	Soot and dust	*Z*	The generation of SO_2_, NO_X_ and SD
GDP	Gross domestic product	*C*	CO_2_
GRA	Grey relation analysis	*X* ^2^	Inputs of the ET stage
SFA	Stochastic frontier analysis	*G*	The emission of SO_2_, NO_X_ and SD
SBM	Slack-based measure	*λ* ^1^	The intensity vector of PP stage
DMU	Decision making unit	*λ* ^2^	The intensity vector of ET stage

## Appendix A

Weak disposability, proposed by Färe et al. [44], considering the potential relationship between desirable outputs and undesirable outputs, has been widely used in the environmental efficiency. To make the model comparable, only the generation and emission of pollutants are considered. The specific model is shown as follows:

(A1) $$\begin{array}{l} E^{w}=\min\frac{1}{2}[\frac{1}{1+3}(\theta +\sum_{p=1}^{3}\varphi_{p})+\frac{1}{3}(\phi_{1}+\phi_{2}+\phi_{3})]\\ \sum_{j=1}^{n}\lambda_{j}^{1}X_{i1j}\leq X_{i10},\;i1=1,\ldots ,m\\ \sum_{j=1}^{n}\lambda_{j}^{1}e_{j}\leq e_{0}\\ \sum_{j=1}^{n}\lambda_{j}^{1}Y_{rj}\geq Y_{r0},\;r=1,\ldots ,s\\ \sum_{j=1}^{n}\lambda_{j}^{1}Z_{pj}=\varphi_{p}Z_{p0},\;p=1,2,3\\ \sum_{j=1}^{n}\lambda_{j}^{1}C_{j}=\theta C_{0}\\ \sum_{j=1}^{n}\lambda_{j}^{2p}X_{j}^{2p}\leq X_{0}^{2p},p=1,2,3\\ \sum_{j=1}^{n}\lambda_{j}^{2p}Z_{pj}\geq Z_{p0},p=1,2,3\\ \sum_{j=1}^{n}\lambda_{j}^{2p}G_{j}^{2p}\leq \phi_{p}G_{0}^{2p},p=1,2,3\\ \sum_{j=1}^{n}\lambda_{j}^{1}Z_{1j}\geq \sum_{j=1}^{n}\lambda_{j}^{21}Z_{1j}\\ \sum_{j=1}^{n}\lambda_{j}^{1}Z_{2j}\geq \sum_{j=1}^{n}\lambda_{j}^{22}Z_{2j}\\ \sum_{j=1}^{n}\lambda_{j}^{1}Z_{3j}\geq \sum_{j=1}^{n}\lambda_{j}^{23}Z_{3j}\\ \sum_{j=1}^{n}\lambda_{j}^{1}=1,\sum_{j=1}^{n}\lambda_{j}^{2p}=1\\ 0\leq \theta ,\varphi_{t},\phi_{1},\phi_{2},\phi_{3}\leq 1\\ \lambda_{j}^{1}\geq 0,\lambda_{j}^{2p}\geq 0 \end{array}$$
